# Oxygenated Eremophilane- and Neolemnane-Derived Sesquiterpenoids from the Soft Coral* Lemnalia philippinensis*

**DOI:** 10.3390/md12084495

**Published:** 2014-08-15

**Authors:** Yun-Jie Xio, Jui-Hsin Su, Yen-Ju Tseng, Bo-Wei Chen, Wangta Liu, Jyh-Horng Sheu

**Affiliations:** 1 Department of Marine Biotechnology and Resources, National Sun Yat-sen University, Kaohsiung 804, Taiwan; E-Mails: yunjie0711@gmail.com (Y.-J.X.); pit0424@yahoo.com.tw (Y.-J.T.); a6152761@yahoo.com.tw (B.-W.C.); 2 Graduate Institute of Marine Biotechnology and Department of Life Science and Institute of Biotechnology, National Dong Hwa University, Pingtung 944, Taiwan; E-Mail: x2219@nmmba.gov.tw; 3 National Museum of Marine Biology and Aquarium, Pingtung 944, Taiwan; 4 Department of Biotechnology, Kaohsiung Medical University, Kaohsiung 807, Taiwan; E-Mail: liuwangta@kmu.edu.tw; 5 Department of Medical Research, China Medical University Hospital, China Medical University, Taichung 404, Taiwan; 6 Graduate Institute of Natural Products, Kaohsiung Medical University, Kaohsiung 807, Taiwan

**Keywords:** *Lemnalia philippinensis*, eremophilane, neolemnane

## Abstract

Five sesquiterpene-related metabolites (**1**–**5**), including two new eremophilane-type compounds, philippinlins C and D (**1** and **2**) and a 4,5-seconeolemnane philippinlin E (**3**), were isolated from the organic extract of a Taiwanese soft coral* Lemnalia philippinensis.* The structures of the new metabolites were determined on the basis of extensive spectroscopic analysis and by comparison of NMR data with those of related metabolites. Compound **3** was suggested to be derived from the neolemnane skeleton.

## 1. Introduction

**Chart 1:**
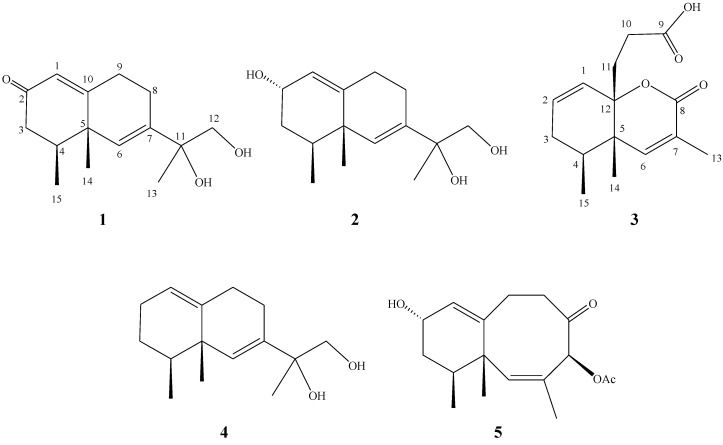
Structures of metabolites **1**–**5**.

In recent years, soft corals have become one of the most prolific sources for the discovery of novel secondary metabolites [1,2]. A variety of sesquiterpenes related to eremophilane- and neolemnane-types, and others have been reported from the Taiwanese soft corals of the genera *Lemnalia* and *Paralemnalia* [3,4,5,6,7,8,9,10]. Our recent study of the chemical constituents of the Lanyu soft coral *L. philippinensis* has yielded ylangene-type sesquiterpenoids, philippinlins A and B [11]. Our continuing investigation of the same collection of this organism again led to the isolation of two new eremophilane-type and one new neolemnane-derived sesquiterpenoids, philippinlins C–E (**1**–**3**), along with two known compounds, 11,12-dihydroxy-6,10-eremophiladiene (**4**) and 4-acetoxy-10-hydroxy-5-oxo-2,8-neolemnadiene (**5**) (Chart 1). The structures of **1**–**5** were elucidated on the basis of extensive spectroscopic analyses and by comparison of the spectral data with those of the related metabolites. The cytotoxicity of metabolites **1**–**5** towards human liver carcinoma (HepG2), human breast carcinoma (MDA-MB231) and human lung adenocarcinoma epithelial cells (A549) was evaluated; however, none of these compounds was shown to exhibit cytotoxicity towards these cancer cell lines in the present study.

## 2. Results and Discussion

Freshly collected soft coral was immediately cooled to −20 °C and kept frozen until extraction. The animal material was extracted exhaustively with EtOAc. The EtOAc extract was fractionated by silica gel column chromatography and the eluted fractions were further separated utilizing normal phase HPLC to afford three new sesquiterpenes (**1**–**3**) together with two known related sesquiterpenes (**4** and **5**). The known sesquiterpenes were readily identified as 11,12-dihydroxy-6,10-eremophiladiene (**4**) [4,5,12] and 4-acetoxy-10-hydroxy-5-oxo-2,8-neolemnadiene (**5**) [4,5], by comparison of their spectral data with those reported in the literatures.

**Figure 1:**
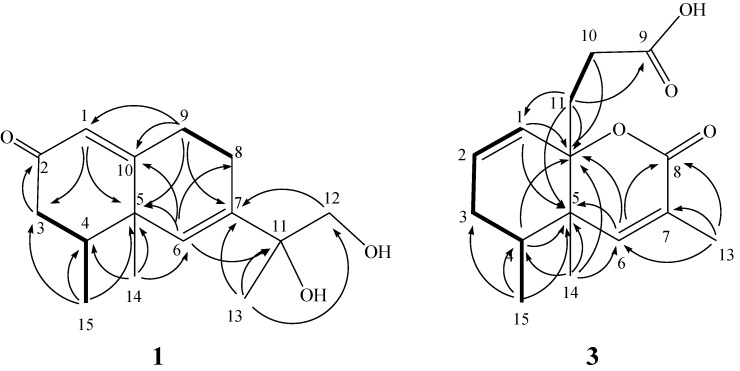
Selected COSY (▬) and HMBC (→) correlations of **1** and **3**.

Philippinlin C (**1**) was obtained as a colorless oil. Its molecular formula was determined as C_15_H_22_O_3_ on the basis of HR-ESI-MS (*m/z* 273.1465, [M + Na]^+^), indicating five degrees of unsaturation. IR absorptions was observed at 3420 cm^−1^, suggesting the presence of hydroxy group in **1**. The ^13^C NMR and DEPT spectroscopic data (Table 1) of **1** revealed the presence of three methyls, four sp^3^ methylenes (including one oxygenated resonating at δ_C_ 68.3), one sp^3^ methine, two sp^2^ methines and five quaternary carbons (including one oxygenated carbons at δ 74.9, two olefinic carbons with resonances at δ 139.4 and 170.1 and one carbonyl carbon δ 199.1). From the required five degrees of unsaturation and the three double bonds known from the above data, a bicyclic sesquiterpene framework of **1** was deduced. The ^1^H NMR data revealed the presence of two olefinic methine protons as one singlet at δ 5.83 and one doublet at δ 5.89, respectively. Furthermore, one oxygenated methylene group (δ 3.62, d, *J* = 11 Hz; δ 3.44, d, *J* = 11.0 Hz) was also designated from the ^1^H NMR signals. The planar structure and all of the ^1^H and ^13^C chemical shifts of **1** were elucidated by 2D NMR spectroscopic analysis, in particular COSY and HMBC experiments (Figure 1). The COSY correlations of H-4 with both H_2_-3 and H_3_-15, and H_2_-8 with H_2_-9 allowed the establishment of two partial structures. The following key HMBC correlations permitted connection of the carbon skeleton: H-1 to C-3 and C-5; H-3 to C-2; H-6 to C-5, C-8, C-10 and C-11; H_2_-9 to C-1, C-5, C-7 and C-10; H_2_-12 to H-7; H_3_-13 to C-7, C-11 and C-12; H_3_-14 to C-4, C-5 and C-6 and H_3_-15 to C-3, C-4 and C-5. Thus, **1** was found to possess two hydroxy groups at C-11 and C-12, two double bonds at C-1/C-10 and C-6/C-7, three methyls at C-4, C-5 and C-11, and a ketone group at C-2. Furthermore, by comparison of the NMR data of **1** with those of **4**, it was found that the ^1^H and ^13^C NMR data of **1** are similar to those of **4**, except the replacement of the CH_2_ in **4** by a ketone group (C=O) in 1. The relative configurations of the two chiral centers at C-4 and C-5 in **1** were elucidated by the following NOE analysis. It was found that H_3_-14 showed NOE interactions with H_3_-15. Thus, assuming the β-orientation of H_3_-14, H_3_-15 should also be positioned on the β face. Based on the above results, the structure of **1** was mostly established. However, the relative configuration of a stereogenic center at C-11 can not be assigned at present stage.

The HR-ESI-MS spectrum of philippinlin D (**2**) exhibited a molecular ion peak at *m/z* 275.1622 ([M + Na]^+^), consistent with the molecular formula C_15_H_2__4_O_3_. Comparison of the NMR data (Table 1) of **2** with that of **1** revealed that the structures of both compounds are similar, with the difference that the ketocarbonyl carbon (C-2) of **1** was replaced by a hydroxy group-bearing methine carbon of **2**. Thus, in the ^13^C NMR spectrum of **1** the signal at δc 199.1 was replaced by a signal at δc 64.1, and in the ^1^H NMR spectrum of **2** the signal at δ_H_ 4.10 (brs) could be attributed to a hydroxyl-bearing methine at C-2. The α-orientation of 2-hydroxy group was determined by comparison of the *J*_1,2_ of **2** with those of elongatols A–B [13], aromatin D and its 2-epimer [14]. Therefore, metabolite **2** was identified as the α-hydroxy derivative at C-2 of the known compound **4**. Still, we are unable to determine the relative configuration of the chiral center C-11 at this time.

**Table 1:** ^13^C and ^1^H NMR spectral data for compounds **1**, **2** and **3**.

1			2		3	
**Position**	**δ_C_**	**δ_H_**	**δ_C_**	**δ_H_**	**δ_C_**	**δ_H_**
	**Multiplicity**	***J* in Hz**	**Multiplicity**	***J* in Hz**	**Multiplicity**	***J* in Hz**
1	124.2 (CH)	5.83 s	121.4 (CH)	5.54 d (4.0)	124.6 (CH)	5.69 d (10.0)
2	199.1 (C)		64.1 (CH)	4.10 br s	132.0 (CH)	5.97 d (10.0, 4.0)
3	42.6 (CH_2_)	2.34 m	36.6 (CH_2_)	1.73 m; 1.65 m	31.0 (CH_2_)	2.08 dt (19.0, 4.0)
						1.80 dd (19.0, 11.5)
4	38.3 (CH)	2.05 m	32.1 (CH)	1.71 m	34.2 (CH)	2.16 m
5	39.9 (C)		38.6 (C)		40.4 (C)	
6	127.5 (CH)		129.1 (CH)	5.88 s	148.3 (CH)	6.30 s
7	139.4 (C)		138.3 (C)		126.0 (C)	
8	26.5 (CH_2_)	2.35 m; 2.12 m	27.5 (CH_2_)	2.22 m; 2.02 m	164.6 (C)	
9	30.7 (CH_2_)	2.57 ddd (13.0, 6.5, 1.5)	29.7 (CH_2_)	2.40 m;	177.1 (C)	
		2.44 ddd (13.0, 5.0, 1.5)		2.20 m		
10	170.1 (C)		147.7 (C)		27.7 (CH_2_)	2.60 m; 2.54 m
11	74.9 (C)		75.0 (C)		28.9 (CH_2_)	2.45 m; 1.73 m
12	68.3 (CH_2_)	3.62 d (11.0);	68.4 (CH_2_)	3.60 d (11.0);	83.4 (C)	
		3.44 d (11.0)		3.40 d (11.0)		
13	23.8 (CH_3_)	1.31 s	23.7 (CH_3_)	1.28 s	16.8 (CH_3_)	1.93 s
14	19.4 (CH_3_)	1.15 s	19.4 (CH_3_)	0.94 s	13.7 (CH_3_)	0.95 s
15	15.2 (CH_3_)	1.06 d (6.5)	15.3 (CH_3_)	0.99 d (6.0)	15.1 (CH_3_)	0.92 d (6.5)

Spectra recorded at 125 MHz in CDCl_3_ for ^13^C NMR and 500 MHz in CDCl_3_ for ^1^H NMR_._

**Chart 2:**
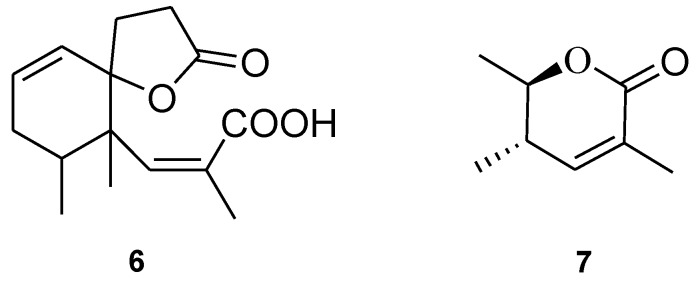
Planar structure of Formula **6** and structure of **7**.

**Figure 2:**
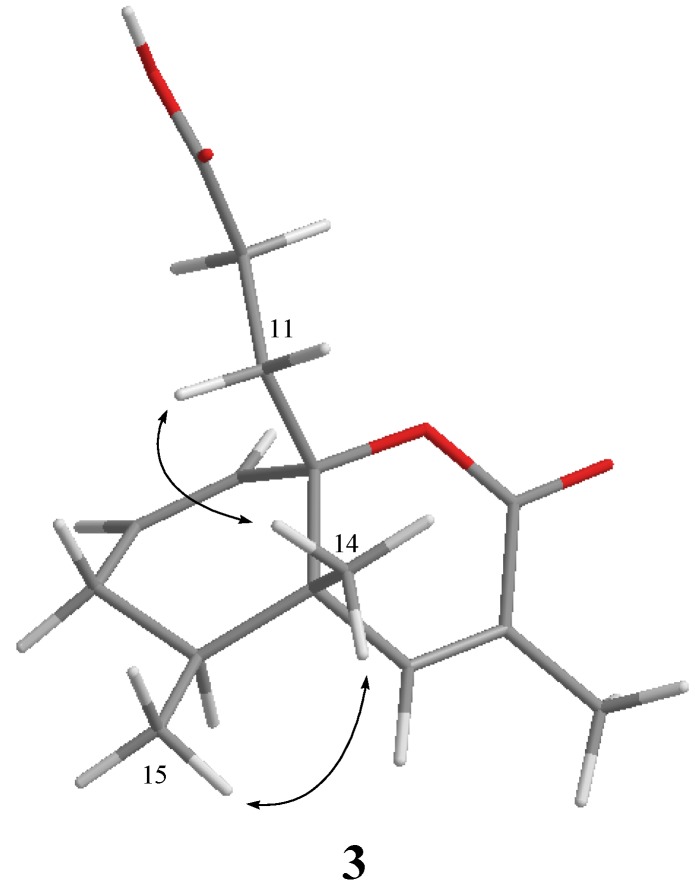
Key NOESY correlations for **3**.

Philippinlin E (**3**) was obtained as a white powder. The molecular formula was determined to be C_15_H_20_O_4_, as deduced from its HR-ESI-MS (*m/z* 287.1257, [M + Na]^+^) and ^13^C NMR data (Table 1). A broad IR absorption at 3402 cm^−1^ indicated the presence of a hydroxy group. The NMR data (Table 1) showed resonances for one trisubstituted double bond (δ_H_ 6.30, s; δ_C_ 148.3 (CH), 126.0 (C)), one 1,1-disubstituted double bond (δ_H_ 5.97 (dd, *J* = 10.0, 4.0 Hz) and δ_H_ 5.69 (d, *J* = 10.0 Hz); δ_C_ 124.6 (CH) and δ_C_ 132.0 (CH)) and two carbonyl carbons (δ_C_ 177.1 and δ_C_ 164.6). To satisfy the six degrees of unsaturation and take into account the presence of two olefinic double bonds and two carbonyl groups, it was assumed that **3** possesses a bicyclic structure. From the COSY spectrum (Figure 1), the partial structures of a proton spin system extending from H-1 to H_3_-15 through H-4 and from H_2_-10 to H_2_-11 could be established, assigning a secondary methyl group at C-4. The molecular framework of **3** was further established by correlations of an HMBC experiment (Figure 1). The two rings and their connectivities to other substituents were elucidated on the basis of the following key HMBC correlations: H_3_-15 to C-3, C-4 and C-5; H_3_-14 to C-4, C-5, C-6 and C-12; H_3_-13 to C-6, C-7 and C-8; H_2_-11 to C-1, C-5, C-9 and C-12; H-1 to C-5 and C-12; H-4 to C-5 and C-12; H-6 to C-5, C-8 and C-12 and H-10 to C-12. Thus, this compound was found to possess two double bonds at C-1/C-2 and C-6/C-7, and two carboxyl groups at C-8 and C-9. Connection of all the above functional groups could lead to the planar structure **3** or an isomeric form 6 (Chart 2). For form **6**, an α,β-unsaturated carboxylic acid and a γ-lacton ring should show IR absorptions at about 1690–1710 and 1760–1770 cm^−^^1^, respectively. However, compound **3** exhibited carbonyl absorption at 1714 cm^−1^ only. Furthermore, 3 was found to have very similar NMR and IR spectroscopic data for the α,β-unsaturated ester moiety in comparison with those of **7** (Chart 2), which has been prepared previously by a stereospecific synthesis [15]. The relative stereochemistry was also confirmed by analysis of the NOESY spectrum (Figure 2). H_3_-14 showed NOE with both H_2_-11 and H_3_-15 but not with H-4. Thus, assuming a β-orientation of H_3_-14, both H_2_-11 and H_3_-15 should be placed on the β face. On the basis of above analysis, the structure of **3** was established.

The skeleton of **3** is new as a natural product, however, **3** has been found previously by treatment of a neolemnane-type compound, 4(S*)-acetoxy,10(S*)-hydroxy,5-oxo,1(S*),12(S*)neolemna-2(*Z*),8-diene, with a methanolic Na_2_CO_3_ solution in air to give an intermediate which should be the unpurified **3**. This unpurified acid was further reacted with diazomethane to give the methyl ester of acid **3** [4]. We are the first group to isolate and characterize **3** from natural sources. Compound **3** might be a natural product, or an artifact from the oxidation of a related neolemane precursor.

**Scheme 1:**
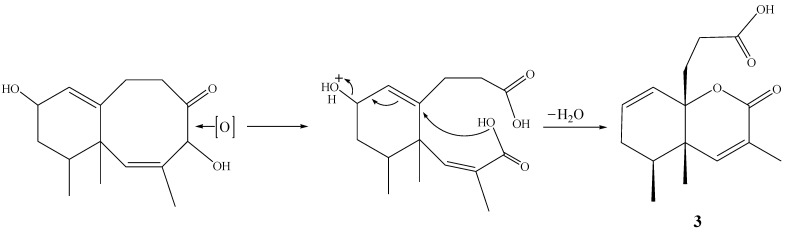
Proposed biosynthetic pathway for **3**.

A plausible biosynthetic pathway of **3** was postulated as shown in Scheme 1. This pathway involves oxidation with ring cleavage of a neolemnane precursor and the subsequent nucleophilic conjugate substitution to afford **3**. The cytotoxicity of metabolites **1**–**5** against the growth of HepG2, MDA-MB231 and A549 carcinoma cells was studied. The results showed that **1**–**5** are not cytotoxic (IC_50_ > 20 μg/mL) toward the above cancer cells. We suggest that further investigation of other bioactivities of these metabolites should be carried out in the future.

## 3. Experimental Section

### 3.1. General Experimental Procedures

Optical rotation values were measured with a Jasco-P1010 digital polarimeter. Infrared spectra were obtained on a Varian Diglab FTS 1000 FT-IR spectrophotometer. NMR spectra were recorded on a Varian Unity INOVA 500 FT-NMR at 500 MHz for ^1^H and 125 MHz for^ 13C or on a Varian 400 MR FT-NMR at 400 MHz for 1^H and 100 MHz for ^13^C in CDCl_3_ at 25 °C. ESIMS and HRESIMS data were recorded on a Bruker APEX II mass spectrometer. Column chromatography was performed on silica gel (230–400 mesh, Merck, Darmstadt, Germany). TLC was carried out on precoated Kieselgel 60 F_254_ (0.25 mm, Merck, Darmstadt, Germany) and spots were visualized by spraying with 10% H_2_SO_4_ solution followed by heating. Normal phase HPLC (NP-HPLC) was performed using a system comprised of a Hitachi L-7110 pump, a Hitachi L-7455 photodiode array detector and a Rheodyne 7725 injection port. A normal phase column (Supelco Ascentis^®^ Si Cat #:581515-U, 25 cm × 21.2 mm, 5 μm) was used for NP-HPLC. Reverse phase HPLC (RP-HPLC) was performed using a system comprised of a Hitachi L-7100 pump, a Hitachi L-2455 photodiode array detector and a Rheodyne 7725 injection port. A reverse phase column (Varian Polaris C18-A, 250 mm × 10 mm, 5 μm) was used for RP-HPLC.

### 3.2. Animal Material

*L. philippinensis*, taxonomically identified by Chang-Feng Dai of National Taiwan University, was collected by hand using scuba off the coast of Lanyu, Taiwan, in August 2008, at a depth of 10–15 m, and stored in a freezer until extraction. A voucher sample was deposited at the Department of Marine Biotechnology and Resources, National Sun Yat-sen University, Taiwan.

### 3.3. Extraction and Isolation

Sliced bodies of the soft coral *L. philippinensis* (0.8 kg, wet wt) were exhaustively extracted with ethyl acetate (EtOAc). The EtOAc extract was evaporated to yield a residue (10.7 g) which was subjected to column chromatography on silica gel by stepwise elution with *n*-hexane-EtOAc mixture and EtOAc-MeOH mixture, to give 25 fractions. Fraction 17, eluting with *n*-hexane–EtOAc (2:1), was further separated by silica gel open column with gradient elution (*n*-hexane–EtOAc, 13:2) to yield 5 subfractions (17A–E). Subfraction 17E was separated by normal phase HPLC using *n*-hexane–EtOAc(2:3) as the mobile phase to afford **1** (1.1 mg), **2** (2.7 mg) and **4** (1.0 mg). Both fractions 18 and 19, eluting with *n*-hexane–EtOAc (1:1–1:2), were combined and further separated by column chromatography over silica gel with gradient elution (*n*-hexane–EtOAc, 7:2) to yield 8 subfractions (18A–H). Subfraction 18D was separated by normal phase HPLC (*n*-hexane–EtOAc, 7:2) to afford **5** (3.5 mg). Subfraction 18G was separated by normal phase HPLC with the elution of *n*-hexane–EtOAc (2:1) to afford **3** (1.9 mg).

Philippinlin C (**1**): colorless oil; [α]^25^_D_ = −175 (*c* 0.1, CHCl_3_); IR (neat) ν_max_ 3420, 2923, 1652 and 1456 cm^−1^; ^1^H and ^13^C NMR data, see Table 1; ESIMS *m*/*z* 273 [M + Na]^+^; HRESIMS *m*/*z* 273.1465 (calcd. for C_15_H_2__2_O_3_Na, 273.1467) (Supplementary Information, Figures S1 and S2).

Philippinlin D (**2**): colorless oil; [α]^25^_D_ = −234 (*c* 0.1, CHCl_3_); IR (neat) ν_max_ 3384, 2922, 2857 and 1373 cm^−1^; ^1^H and ^13^C NMR data, see Table 1; ESIMS *m*/*z* 275 [M + Na]^+^; HRESIMS *m*/*z* 275.1622 (calcd. for C_15_H_24_O_3_Na, 275.1623) (Supplementary Information, Figures S3 and S4).

Philippinlin E (**3**): white powder; mp 124 °C; [α]^25^_D_ = −134 (*c* 0.2, CHCl_3_); IR (neat) ν_max_ 3402, 2940, 1714 and 1371 cm^−1^; ^1^H and ^13^C NMR data, see Table 1; ESIMS *m*/*z* 287 [M + Na]^+^; HRESIMS *m*/*z* 287.1257 (calcd. for C_15_H_2__0_O_4_Na, 287.1259) (Supplementary Information, Figures S5 and S6).

### 3.4. Cytotoxicity Testing

Cell lines were purchased from the American Type Culture Collection (ATCC). Cytotoxicity assays of compounds **1**–**5** were performed using the MTT [3-(4,5-dimethylthiazol-2-yl)-2,5-diphenyl-tetrazolium bromide] colorimetric method [16,17].

## 4. Conclusions

Two new eremophilane-type compounds philippinlins C and D (**1** and **2**) a new 4,5-seconeolemnane philippinlin E (**3**), along with two known compounds, 11,12-dihydroxy-6,10-eremophilaiene (**4**) and 4-acetoxy-10-hydroxy-5-oxo-2,8-neolemnadiene (**5**), were discovered from the soft coral* L*. *philippinensis*. The molecular skeleton of **3** was discovered for the first time from natural sources. 

## Supplementary Files

Supplementary File 1
