# Supplementary material for: Oxygenated Eremophilane- and Neolemnane-Derived Sesquiterpenoids from the Soft Coral Lemnalia philippinensis
Source: Mar Drugs. 2014 Aug 15;12(8):4495–503. doi: 10.3390/md12084495 (PMC4145327; doi:10.3390/md12084495)

Supplementary Information

Figure S1. ^{1}H NMR spectrum of **1** in $CDCl_3$.

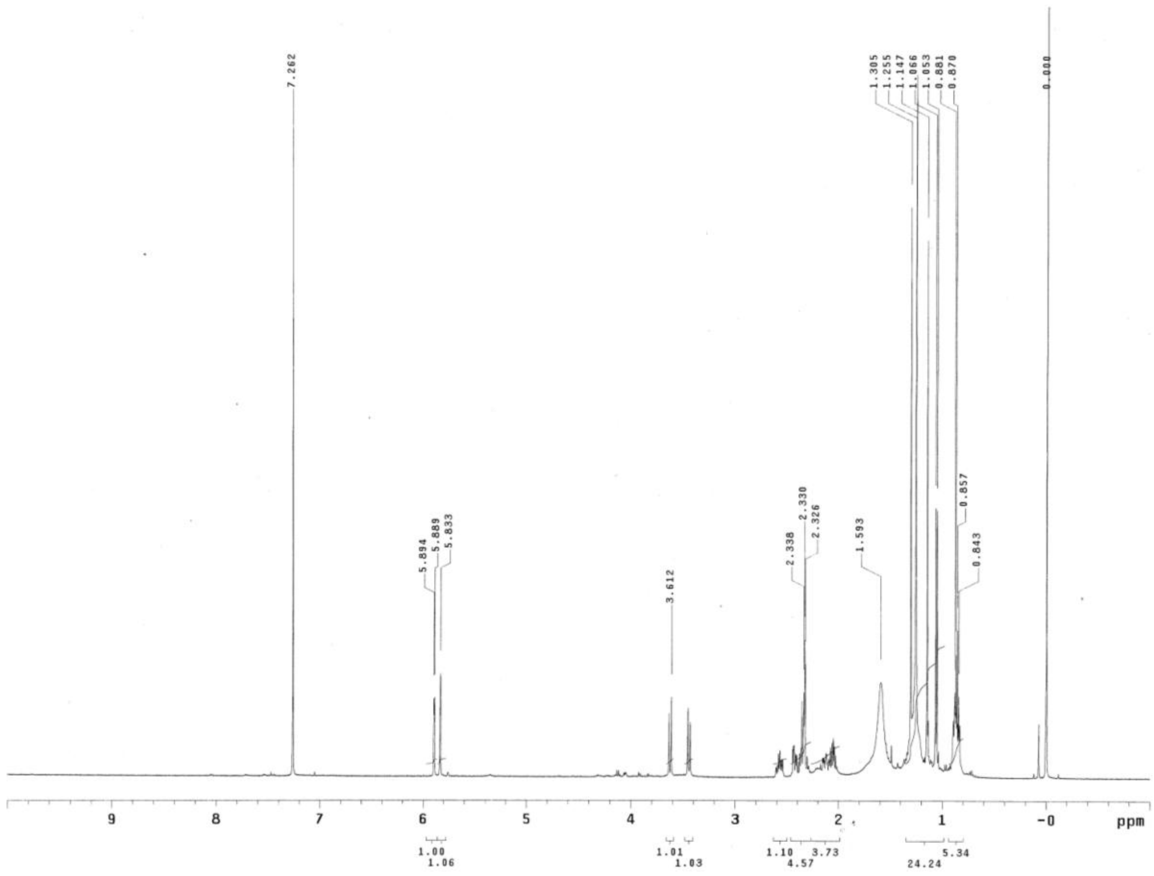

Figure S2. ^{13}C NMR spectrum of **1** in $CDCl_3$.

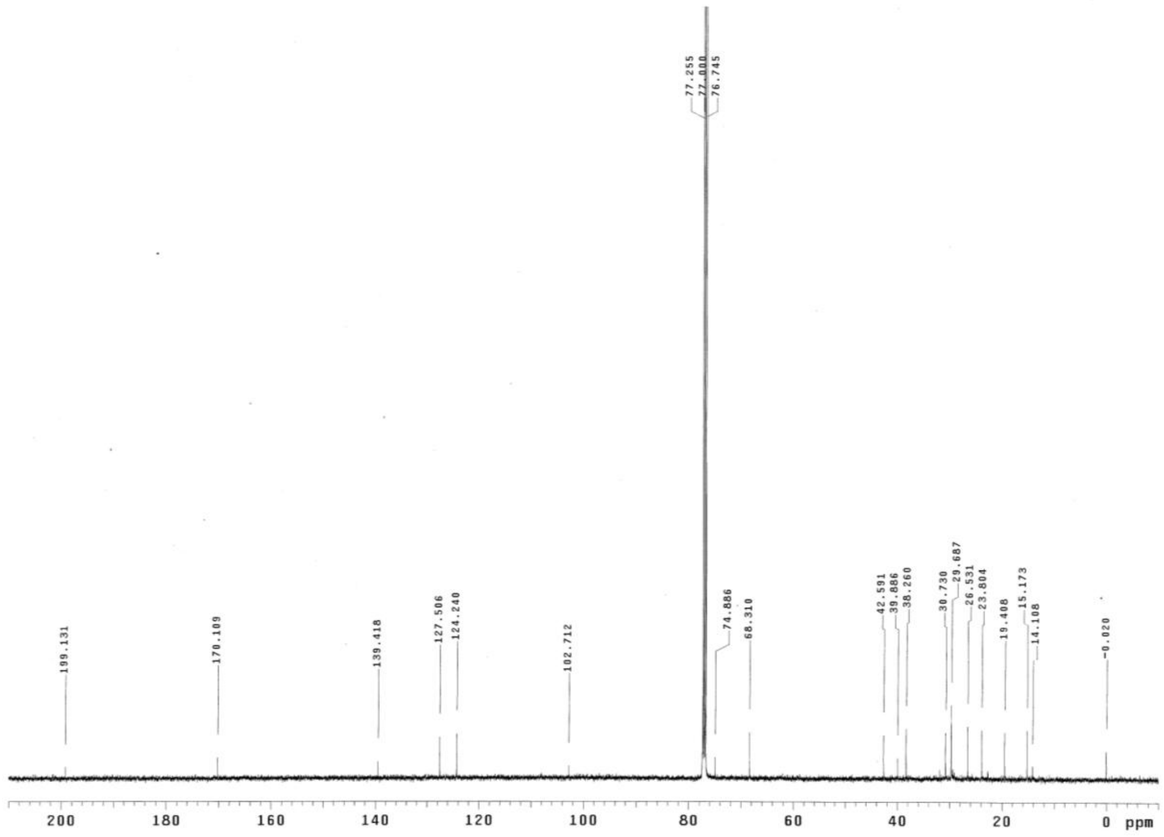

Figure S3. ^{1}H NMR spectrum of **2** in $CDCl_3$.

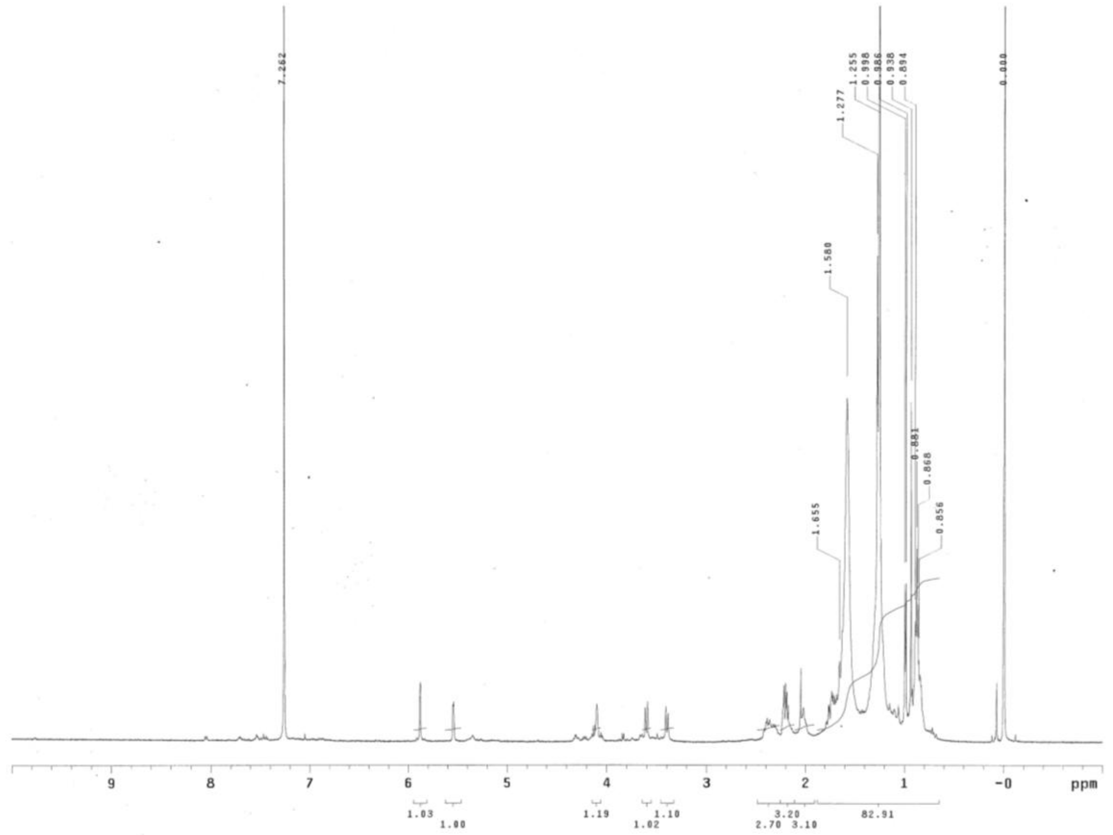

Figure S4. ^{13}C NMR spectrum of **2** in $CDCl_3$.

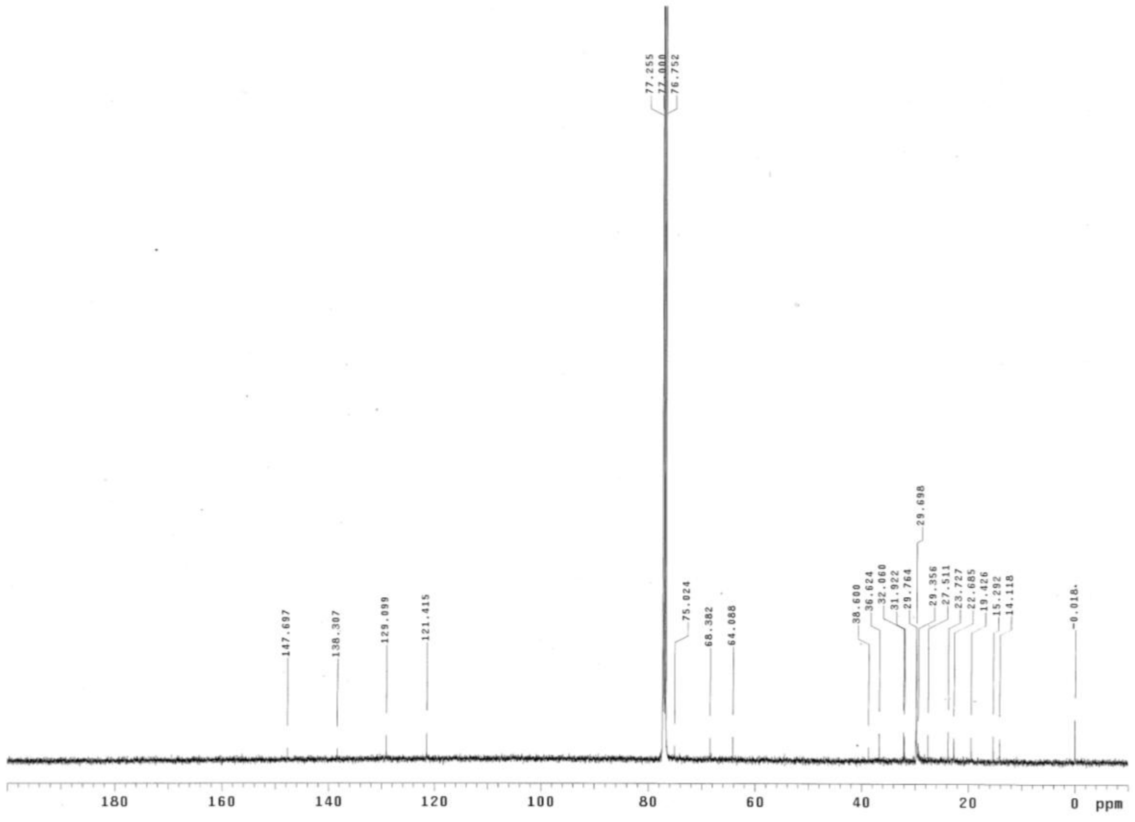

Figure S5. ^{1}H NMR spectrum of **3** in $CDCl_3$.

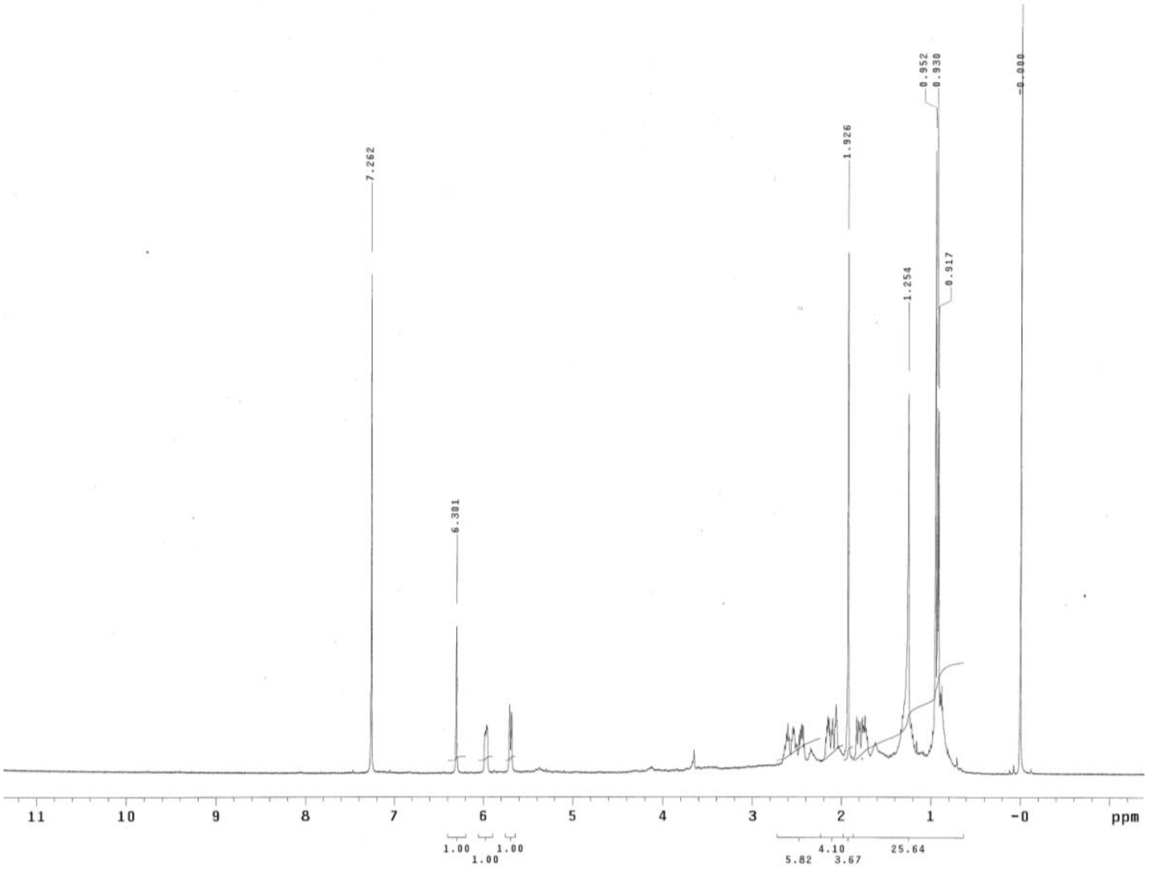

Figure S6. ^{13}C NMR spectrum of **3** in $CDCl_3$.

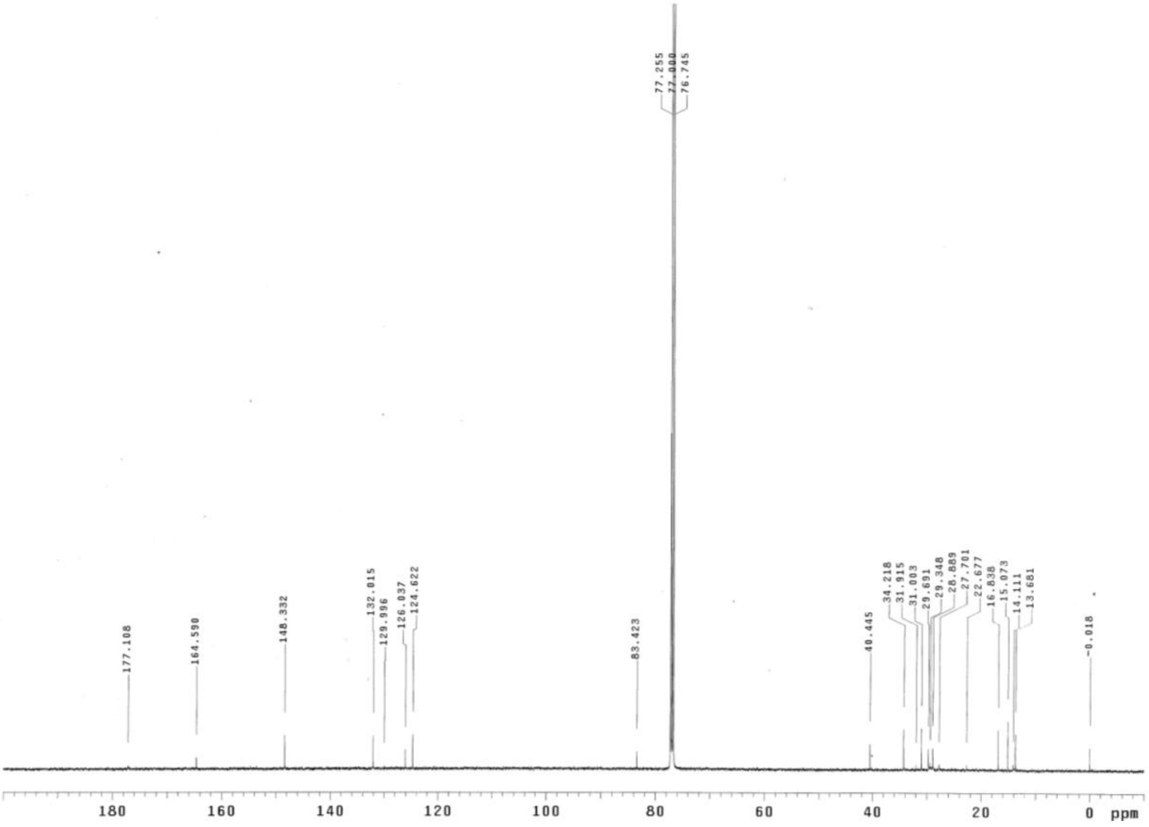

Supplement: Supplementary File 1 [file marinedrugs-12-04495-s001.pdf]
